# Cerebral blood flow changes during aging process and in cognitive disorders: A review

**DOI:** 10.1177/19714009211002778

**Published:** 2021-03-22

**Authors:** Naghmeh Mokhber, Aidin Shariatzadeh, Abolfazl Avan, Hamidreza Saber, Golnaz Shojaeian Babaei, Gary Chaimowitz, M. Reza Azarpazhooh

**Affiliations:** 1 Department of Psychiatry, Western University, Canada; 2 Department of Psychiatry and Neuropsychiatry, Mashhad University of Medical Sciences, Iran; 3 Stroke Prevention and Atherosclerosis Research Centre, Robarts Research Institute, Canada; 4 Department of Public Health, Mashhad University of Medical Sciences, Iran; 5 Department of Neurology, Wayne State University School of Medicine, USA; 6 Department of Psychiatry and Behavioural Neurosciences, McMaster University, Canada; 7 Department of Clinical Neurological Sciences, Western University, Canada

**Keywords:** Cerebrovascular circulation, neurovascular coupling, functional imaging, cognitive disorders, neuronal loss, aging

## Abstract

We aimed to summarize the available evidence on cerebral blood flow (CBF) changes in normal aging and common cognitive disorders. We searched PubMed for studies on CBF changes in normal aging and cognitive disorders up to 1 January 2019. We summarized the milestones in the history of CBF assessment and reviewed the current evidence on the association between CBF and cognitive changes in normal aging, vascular cognitive impairment (VCI) and Alzheimer’s disease (AD). There is promising evidence regarding the utility of CBF studies in cognition research. Age-related CBF changes could be related to a progressive neuronal loss or diminished activity and synaptic density of neurons in the brain. While a similar cause or outcome theory applies to VCI and AD, it is possible that CBF reduction might precede cognitive decline. Despite the diversity of CBF research findings, its measurement could help early detection of cognitive disorders and also understanding their underlying etiology.

## Introduction

Normal neuronal cell activity and brain function need a simultaneous increase in cerebral blood flow (CBF) in response to an increased energy demand. There is controversial evidence regarding the association between age-related CBF changes and the development and progression of cognitive impairment and dementia. Previous reviews of CBF studies and dementia focused on CBF regulation and neurovascular dysfunction,^1^ cardiovascular determinants of CBF in normal aging^2^ and regional changes in dementia subtypes, and discussed specific CBF assessment techniques.^3–5^ The main goal of this review is to provide a summary of age-related CBF changes compared with neurocognitive diseases.

## Methods

For this narrative review, we searched PubMed and bibliographies of relevant articles, as well as book chapters up to 1 January 2019. We used Medical Subject Headings and entry terms related to cerebral circulation, aging and cognition and dementia to identify all relevant published studies. We summarized evidence on the history and utility of CBF in cognition research.

## Results

### Milestones in CBF study and measurement techniques

In 1870, Adolf Eugen Fick defined blood flow as the amount of oxygen used by an organ, such as the heart, over a certain time period.^6^ In the early 1880s, Angelo Mosso, an Italian physiologist, measured CBF by recording changes in brain pulsations in a 37-year-old farmer with a large skull fracture.^7^ He introduced the first medical device (plethysmograph) to measure CBF change. He was able to record and quantify brain volume changes in response to cognitive activities. Mosso’s method, in fact, is still the basis of current functional imaging studies, assessing CBF after cognitive tasks. In 1890, Roy and Sherrington^8^ realized that neuronal activities in the brain were associated with simultaneous changes in blood flow, the idea that led to the concept of ‘coupling’ in functional imaging studies. In 1928, Forbes^9^ used the cranial window to measure pial vessel flow, which later improved our understanding of the pial microcirculation and vascular physiology.^10^ In 1943, Dumke and Schmidt^11^ used an invasive quantitative measurement of CBF with the bubble-flow meter in a macaque monkey.

For the next 60 years, after the Mosso’s report, neuroscientists measured CBF in subjects with skull defects. In the 1940s, Kety and Schmidt^12^ used low concentrations of nitrous oxide to determine CBF in conscious human subjects. Despite its poor spatial and temporal resolution, this method remains as an important resource to study the physiology of cerebral circulation. With some modification in the Kety–Schmidt method, Niels Lassen^13^ managed to measure regional CBF (rCBF) with radioactive krypton. In 1955, focal brain circulation in cats was evaluated with trifluoroiodomethane as a tracer.^14^ Later, radionuclide techniques provided a more accurate evaluation of the regional functional activity via measurement of regional cerebral glucose consumption. Metabolic markers, including [14C] deoxyglucose and [18F] fluorodeoxyglucose (FDG), were employed in single photon emission computed tomography (SPECT) and positron emission tomography (PET) scan. In the 1980s, the transcranial doppler (TCD), as a non-invasive method, was developed^15^ and has been frequently used in CBF studies. In 1990, Seiji Ogawa showed the difference in magnetic properties of oxygenated and deoxygenated hemoglobin.^16^ This was a revolution in modern functional magnetic resonance imaging (MRI) and led to a technique known as blood oxygenation level-dependent or (BOLD) contrast. BOLD is a marker of neuronal activity and a good representative of CBF, cerebral blood volume (CBV) and cerebral metabolic rate of oxygen (CMRO2).^17^

In 1994, Kashimada et al.^18^ measured CBF with two-dimensional cine phase-contrast MRI in 24 healthy subjects. In 2002, Spilt et al.^19^ confirmed the accuracy of phase-contrast MRI in measuring total CBF in healthy individuals. The arterial spin labeling technique, including continuous and pseudo-continuous measurement, was also developed as a minimally invasive method, requiring no exogenous tracers, which makes it appropriate for dynamic CBF measurement in healthy individuals.^20–22^ Currently, PET using radiolabeled water (15O-water) is one of the best methods to measure CBF.^23^ CBF measurement with H215O‐PET needs an arterial input function (AIF). AIF requires continuous arterial blood sampling. However, a new technique using simultaneous PET and phase‐contrast MRI (PC‐MRI) is able to quantify CBF without blood sampling.^19^,^24^

In summary, since Mosso’s era, CBF measurement techniques have greatly improved from direct observations to indirect metabolic consumption measurement, and are still being used in daily practice and clinical research.

### CBF changes in normal aging

Scientists have frequently swung their descriptions of dementia from ‘vascular hardening’ to ‘Alzhemirization’ and vice versa.^25^ In the late nineteenth and mid-twentieth centuries, there was a common belief that brain artery stiffness due to aging could cause chronic ischemia and brain failure.^26^ This theory resulted in an excessively global use of vasodilators in order to enhance CBF,^26^ which was later abandoned. In the mid-1970s, rCBF assessment showed a normal vasodilatation in response to changes in CO_2_ concentration in the brains of patients with primary degenerative dementia,^27^ indicating that there was no considerable vascular hardening and chronic ischemia. In 1981, Frackowiak et al. did not find an increase in global oxygen extraction ratio (as expected in ischemia) in PET scans of subjects with neurodegenerative dementia. Later, AD – which used to be regarded as a rare presenile disease – became synonymous with dementia, in the way that vascular hardening with low CBF had been.^25^ This paradigm shift with a bias toward Alzheimization or vascular hardening has remained a challenging issue in the pathophysiology of dementia. Nevertheless, blood flow studies may help in understanding pathophysiological processes of neurodegenerative and vascular cognitive disorders and elucidate their similarities and differences.

Learning patterns and rates of age-related blood flow change is the first step in CBF studies of cognitive disorders. Although CBF might remain unchanged or minimally diminished during normal aging,^28^,^29^ most studies found a gradual decline,^30^ ranging from 3.9 mL/min^18^ to 4.8 mL/min (0.52%) blood flow decline per year.^31^ An age-related CBF reduction occurs in the brain cortex (0.45% to 0.74% decline per year in gray matter) with only minimal changes in subcortical regions (0.3% decline per year in white matter).^21^,^30^,^32–34^ The normal blood supply of the brain tissues varies from 20 mL/100g/min in white matter to 70 mL/100g/min in gray matter.^35^

The reasons for age-related CBF reduction are still a matter of debate. There is a negative correlation between global CBF and subjective rates of cortical atrophy with aging.^28^,^36^ Some studies have shown that this reduction might be due to progressive neuronal loss, diminished neuronal activity and decreased synaptic density of brain neurons.^33^,^37^,^38^ Age-related CBF changes should be discussed according to the neurovascular system, from the smallest neurovascular unit to large cerebral arteries. There is a significant pressure gradient difference across cerebral arteries. The base of the brain (i.e., vascular centrencephalon) is supplied by relatively short and straight arteries. These are end arteries without substantial collateral supply, transmitting blood pressure directly to small vessels. In contrast, in the cerebral convexity, blood flow transfers from large and medium-sized cerebral vessels, passing through long arteries with many collateral to cerebral peripheral beds.^39^,^40^ In a normotensive person, a blood pressure of 117/75 mm Hg in brachial arteries is accompanied by a pressure of 59/38 mm Hg in small parietal arterioles.^39^ Such a low pressure would induce white matter lesions (WMLs), which explains the correlation between a low CBF and white matter changes.^3^ Further, high CBF is associated with less severe WMLs.^41^ According to the Rotterdam Study,^42^ those with a higher middle cerebral artery velocity have a lower chance of hippocampus atrophy and dementia. A study on 7700 brain images from the Alzheimer’s Disease Neuroimaging Initiative study showed a possible causative effect of CBF change on cognitive decline.^43^ It suggests that intra-brain vascular dysregulation is an early pathological event during the development of late onset AD. In addition to CBF changes related to hypotension, WMLs may happen due to dysfunction of the blood–brain barrier (BBB).^44^ Wong et al.^44^ showed a significant decrease in CBF with an increase in leakage volume in perilesional zones close to WMLs using dynamic susceptibility contrast and dynamic contrast-enhanced MRI in 27 cases with lacunar stroke or VCI. The presence of such leaky vessels may be explained by regional BBB permeability. As BBB and CBF are both regulated in the neurovascular unit (NVU), WMLs may be due to deterioration of this unit.^1^,^44^ In addition, it is hypothetically possible to have a reciprocal relationship between rCBF changes with autonomic control, modulating the cardiovascular system; i.e. insula^45^ and cingulate gyrus^46^ and further total and regional CBF changes.

Age-related cardiovascular dysfunction can change CBF.^2^ The rise in pulsatile hemodynamic stress to the brain may also play a role in age-related blood flow and cognitive changes. The aging process in large arteries, such as the aorta, may increase vascular stiffness, but reduce vascular compliance, which transfers more pulsatile energy to the brain.^47^ Theoretically, such a pulsatile energy might lead to cerebral microvascular disease, and consequently cognitive impairment.^48^ However, this association was not confirmed in a TCD study.^49^ With aging, a dramatic increase can also be seen in the prevalence of cardiovascular diseases. For example, CBF and its regulation could also be influenced by common comorbidities in the elderly, such as hypertension^50^ or antihypertensive medications.^51^,^52^

### CBF changes in vascular and neurodegenerative dementia

**Figure 1.**
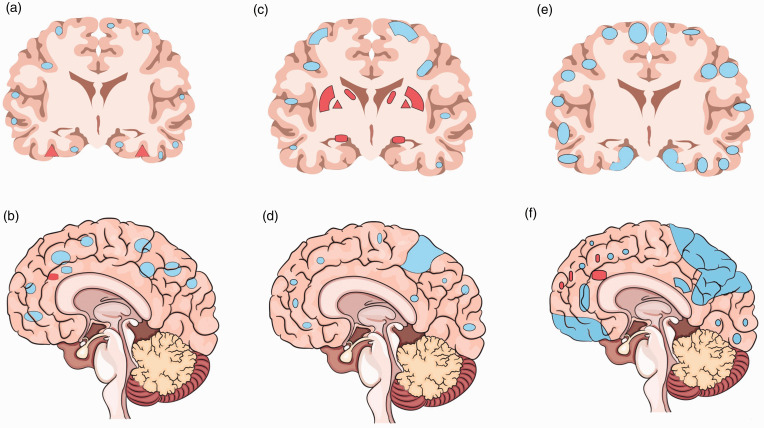
Stepwise pattern of cerebral blood flow changes in Alzheimer's disease. (a) Sagittal view and (b) coronal view in asymptomatic APO ε4 carriers; (c) sagittal view and (d) coronal view in those with mild cognitive impairment; and (e) sagittal view and (f) coronal view in those with frank dementia.

**Figure 2.**
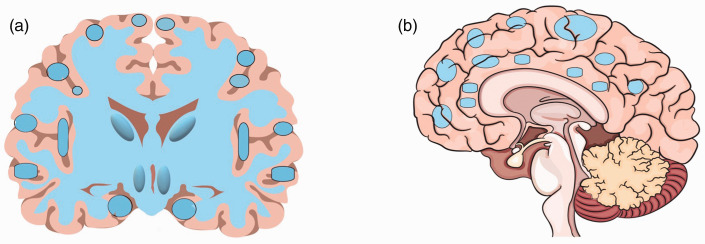
Cerebral blood flow reduction in patients with vascular cognitive impairment. (a) Coronal view and (b) sagittal view.

Compared to age-related CBF changes with trivial changes in subcortical areas, AD and neurodegenerative dementias have more specific regional changes in rCBF (Table 1) that could be related to underlying pathophysiology of the disease. While it can be debated that regional hypoperfusion in dementia reflects diminished demand due to brain atrophy and neuronal loss, several studies suggest that CBF changes may contribute to brain lesions, and thus precede cognitive impairments. Similar to clinical stages of AD, CBF may change in a stepwise pattern (Figure 1): asymptomatic stage (in APO ε4 carriers)^53^ and preclinical (prior to amyloid-β accumulation or brain atrophy).^54–57^ Compared to healthy volunteers, in cases with mild cognitive impairment (MCI)/early AD, a selective CBF reduction can be found in precuneus and posterior cingulate gyrus.^58^,^59^ While in MCI a compensatory high CBF may happen in several areas (i.e., hippocampus, amygdala, caudate, putamen and globus pallidus), such CBF changes may be negative or only found in limited (frontal lobe, anterior cingulate gyrus) brain regions of AD patients (Figure 2). During the transitional stage from MCI to AD, a decreased rCBF develops in other brain areas, including parietotemporal, parahippocampal gyrus, hippocampus, entorhinal cortex, frontal cortex and occipital cortex.^54–57^ Lower CBF in the posterior brain regions,^60^ including parietal^61^ and parieto-temporal lobes, is a predictor of a rapid conversion from MCI to AD.^62^

**Table 1.** Blood flow changes in aging and dementias.

Aging/cognitive disorders		Blood flow changes and brain regions	Other findings	Measurement techniques
Aging		Reduced CBF in the cortex of lateral occipital, cingulate, precuneus,^32^ temporal,^32^,^34^ parietal,^32^,^34^ insular and frontal lobes^32,^^33^	No CBF change in subcortical areas^32^	ASL-MRI;^32^ PET;^33^ SPECT^34^
Vascular cognitive impairment		Multiple regional CBF reduction with a posterior–anterior gradient, sparing occipital lobe;^63–66^ extensive white matter involvement with a tendency toward subcortical circuit^44^	NVU dysregulation due to a combination of hypoperfusion and BBB permeability^44^	SPECT;^63,^^64^ ASL-MRI;^65^ PET;^66^ DCE/DSC-MRI^44^
Alzheimer’s disease	Asymptomatic phase	Regional blood flow changes in asymptomatic middle-aged adults with a maternal history of AD^53^ and APOε4 carriers^67^	CBF difference between older and younger APOε4 carriers^67^	ASL-MRI^53,^^59^,^67^
	MCI	Intra-brain vascular dysregulation as early pathological findings with disease development.^43^ Reduced CBF in the occipital,^68^ angular gyrus, temporal,^62^,^68^ posterior cingulate gyrus, cuneus,^69^ parietal^62,^^70^ and frontal lobes^62^	Compensatory CBF increment in hippocampus, amygdala, caudate, putamen and globus pallidus;^69^ CMBs in 25% of cases^71^	ASL-MRI;^68–70^ GE-MRI;^62^ 2D phase-contrast MRI;^54^ SW-MRI^71^
	Dementia	Regional CBF reduction beyond the MCI regions with a prominent decline in the medial temporal lobe,^72^ posterior cingulate gyrus,^69^,^70^ and inferior parietal cortex^69^	Limited compensatory CBF increment in the anterior cingulate gyrus;^69^ lobar CMBs (78% of cases)	ASL-MRI;^57,^^58^,^68–70^,^73^ GE-MRI;^62^ SPECT;^72,^^74^ PET;^58,^^66^ 7-tesla MRI^75^
Amyotrophic lateral sclerosis		Generalized CBF reduction (whole cortex and subcortical areas);^76^ regional CBF reduction in the frontal and parietal lobes^77^	CMBs in motor cortex^78^	ASL-MRI;^77^ CT;^76^ MRI^78^
Frontotemporal dementia		Reduced CBF in the frontal lobe^73^	Increased CBF in medial parietal, posterior cingulate and precuneus^73^	ASL-MRI
Huntington's disease		Reduced CBF in the sensorimotor paracentral, temporal, occipital, postcentral gyrus and insula^79^		ASL-MRI
Lewy body dementia		Reduced CBF in the parietal, temporal and occipital lobes;^74^ occipital hypoperfusion^74,^^80^		SPECT;^74^ Radio pharmacological techniques^74,^^80^
Multiple sclerosis		Reduced CBF in both white^81,^^82^ and gray matter^82^	Increased BBB permeability;^81^ impaired cerebrovascular reactivity^83^	DCE-MRI;^81^ DSC-MRI;^82^ ASL-MRI^83^
Parkinson’s disease		Reduced CBF in the parietal, occipital, frontal, cuneus,^84^ supramarginal gyrus,^85^ precuneus, temporal, cingulate^84,^^85^ and subcortical areas (thalamus and caudate)^84^	CMBs in both white and gray matter;^86,^^87^ impaired whole brain cerebrovascular reactivity^88^	T2-MRI and SWI-MRI;^86,^^87^ ASL-MRI^84,^^85^,^88^
Progressive supranuclear palsy		Reduced CBF in the frontal lobe^89^		SPECT

Abbreviations: 2D: two-dimensional; ASL-MRI: arterial spin labeling magnetic resonance imaging; BBB: blood–brain barrier; CBF: cerebral blood flow; CMBs: cerebral microbleeds; CT: computed tomography; DCE-MRI: dynamic contrast-enhanced magnetic resonance imaging; DSC-MRI: dynamic susceptibility contrast magnetic resonance imaging; GM: gray matter; MCI: mild cognitive impairment; MRI: magnetic resonance imaging; NVU: neurovascular unit; PET: positron emission tomography; rCBF: regional cerebral blood flow; SPECT: single photon emission computed tomography; SWI: susceptibility-weighted imaging; WM: white matter.

Compared to AD, in VCI, various brain regions are involved with a gradient from posterior to anterior brain regions (Table 1, Figure 2).^63^ Blood flow may change in both gray matter^63–66^ and white matter,^66^ or merely white matter with a tendency toward extensive CBF changes in subcortical circuits^44^ (Figure 1). While regional CBF changes could be observed with aging (Table 1), such stepwise patterns of CBF changes do not happen in VCI. This important finding might provide opportunities for interventions prior to clinical symptoms.

## Discussion

Knowledge about the patterns of CBF changes in cognitive decline and normal aging may provide a useful tool to assess individuals at risk and to identify the pathophysiology of cognitive changes. In the current review, we summarized different contributors of age-related CBF changes. They can be classified into non-modifiable age-related changes (i.e., cortical atrophy with aging^28^,^36^ with changes in synaptic density of brain neurons^33^,^37^,^38^) to modifiable cardiovascular determinants of CBF (i.e., blood pressure, changes in cerebral arteries and NVU). Such knowledge about the pattern of CBF in the elderly emphasizes the opportunity for prevention by controlling risk of cardiovascular diseases.^2^

The pattern of CBF changing could help differentiate between CBF changes in age-related and neurocognitive diseases. There is a stepwise pattern of CBF changes in AD. The pattern of regional CBF changes follows an almost similar model with progression of AD from asymptomatic phases to dementia. This finding has clinical implications including identifying cases with possible AD. This result may also guide clinicians and scientists in selecting cases for studies on changes of the baseline CBF.

## Conclusion

In the current review, we summarized CBF changes that can be seen in different neurocognitive changes that could be matched with underlying diseases. However, it is not still clear that CBF changes in aging and neurocognitive diseases are the cause or outcome of neuronal loss. In AD, the stepwise changes in total and regional CBF are suggestive of a causal role for CBF changes in the pathophysiology of the disease. This theory needs to be tested for other neurocognitive diseases. Knowledge regarding CBF changes in different diseases and aging has clinical implications in understanding the pathophysiology of diseases, their diagnosis and prevention.

## Highlights

- Patterns of cerebral blood flow correspond with cognitive disorders.
- Cerebral blood flow usually remains unchanged or minimally declines during aging.
- Cerebral blood flow changes may precede cognitive decline.
- Cerebral blood flow differs from asymptomatic AD to dementia.
- In vascular cognitive impairment, most brain regions show declined blood flow.

## Disclosures

The authors have no competing interests to declare.
